# Thermoregulation in the appendix of the *Sauromatum guttatum* (Schott) inflorescence

**DOI:** 10.1186/s40529-014-0068-0

**Published:** 2014-09-26

**Authors:** Hanna Skubatz

**Affiliations:** 2023 120th Ave NE, Suite S128, Bellevue, 98005 WA USA

**Keywords:** Appendix, Aspirin, Arum italicum, 2,6-Dihydroxybenzoic acid, Salicylic acid, Sauromatum guttatum, Thermoregulation

## Abstract

**Background:**

Three phenolic compounds are capable of activating the process that simultaneously leads to temperature rise and odor-production in the *Sauromatum* appendix. These compounds are salicylic acid, aspirin, and 2,6 dihydroxybenzoic acid. The objectives of the present study were to examine the effect of various concentrations of the these inducers on the temperature rise and to study the effect of mitochondrial inhibitors (KCN and SHAM) and an uncoupler (DNP) on the temperature rise.

**Results:**

In sections of the *Sauromatum* appendix two successive temperature rate maxima were detected in the presence of the three inducers. Two temperature maxima were also detected in appendices of intact inflorescences. The temperature profiles demonstrated a considerable variability within sections of one appendix in both magnitude and time of reaching a peak. When the *Sauromatum* temperature decreased it returned either to the same temperature baseline or to a slightly different baseline. The temperature rise was blocked by KCN (20 mM) and SHAM (40 mM) alone or when added together. DNP, an uncoupler, at 2.5 mM also blocked the rise in temperature. The thermogenic inducers also triggered a temperature rise in *Arum* appendix.

**Conclusions:**

The presence of two rate maxima may indicate different heat-generating sources. The blockage of the temperature rise in the presence of KCN or SHAM implies that the activity of the cyanide-resistant and -sensitive pathways is required for generating heat. The variability in temperature profiles maybe related to changes in cellular control factors. This study provides the basis for investigating thermoregulation in plants.

**Electronic supplementary material:**

The online version of this article (doi:10.1186/s40529-014-0068-0) contains supplementary material, which is available to authorized users.

## Background

One striking example of a thermogenic plant is *Sauromatum guttatum* (the voodoo lily). On D-day, the day of inflorescence opening, the *Sauromatum* appendix (a ~20 cm-long, slender organ that comprised the upper part of the inflorescence) becomes hot, reaching 32°C above room temperature (Skubatz et al. [1991]). The heat-loss reaches a value of 1 mWatt/mg DW (Skubatz and Meeuse [1993]). The temperature rise correlates with a high rate of respiration (Meeuse [1966], [1975]). The heat facilitates the release of odorants that attract pollinators.

The current concept on the rising temperature in the *Sauromatum* appendix is that heat is generated by the activity of AOX, which is present in the *Sauromatum* mitochondria as well as in mitochondria of many non-thermogenic plants. Basically, AOX activity acts as an energy overflow of the Cyt pathway. When the Cyt pathway is restricted or saturated, AOX becomes active and since it does not generate a proton-motive force, the energy is released as heat (Elthon and McIntosh [1986]; Wagner et al. [2008]). On D-day, the amount of COX in the *Sauromatum* mitochondria is low, and the amount of AOX is high. Therefore, the electron flow to AOX is intense (Elthon et al. [1989]). In non-thermogenic plants, AOX is involved in the protection against oxidative stress (Wagner and Moore [1997]). In some thermogenic plants, the mitochondrial UCPs generate heat by uncoupling the electron transport from phosphorylation (Borecky et al. [2001]; Ito-Inaba et al. [2008]; Smith et al. [2004]; Zhu et al. [2011]).

Salicylic acid, ASA, and 2,6-DHBA activate the temperature rise as well as odor-production in the *Sauromatum* appendix (Raskin et al. [1989]). Treatment of pre D-Day (3 to 1 d prior to heat-production) appendix sections with SA at concentrations higher than 1 μM induced significant increases in temperature (Skubatz et al. [2013]). Salicylic acid effects are not confined only to the spadix of arum lilies. The acid has many roles in non-thermogenic plants and is considered a plant hormone (Vlot et al. [2009]).

In the present study the temperature rise in sections of the *Sauromatum* and *Arum* appendices in the presence of the three inducers was recorded. The temperature profiles revealed two successive temperature rates that fluctuated in regard to their maximum values and the time it took to reach a maximum. Two temperature maxima were also detected in appendices of intact *Sauromatum* inflorescences. Surprisingly, application of KCN alone blocked the rise in temperature of the *Sauromatum* appendix whereas SHAM significantly but not completely blocked the rise in temperature. Application of KCN and SHAM together blocked completely the temperature rise. DNP, an uncoupler, also unexpectedly blocked the rise in temperature. These results provide evidence that the temperature rise may involve AOX and Cyt pathways. The blockage of the temperature rise by DNP, an uncoupler that dissipates the proton gradient and increases the electron flux via the Cyt pathway suggests that *in vivo* the Cyt pathway is required for heat-production and that UCPs may not be involved in this process in the *Sauromatum* appendix.

## Methods

### Chemicals

Dinitrophenol, KCN, SHAM, 2,6-DHBA, and ASA were purchased from Sigma-Aldrich, St. Louis, MO. SA was purchased from Thermo Fisher Scientific Inc., Pittsburgh, PA. Salicylic acid, ASA, and 2,6-DHBA were dissolved in distilled water. Salicylhydroxamic acid was dissolved in 100% DMSO to produce 1 M stock solution.

### Plant materials

Corms of *Sauromatum guttatum* were kept at 4°C and the inflorescences were allowed to develop under a 15/9 day/night cycle at room temperature. When an inflorescence matures the base of its spathe becomes swollen and its color changes to burgundy. These characteristics appear 3 d prior to heat-production (pre D-day stage). At this stage, application of an inducer to sections of the appendix triggers heat-production.

*Arum itallicum* was grown outdoors at the University of Washington. *Arum* inflorescences were also cut at pre D-day stage, prior to inflorescence opening and heat-production. The thermogenic response of sections of the *Arum* appendix in the presence of inducers was recorded.

The *Sauromatum* appendix is ~5 times longer than the *Arum appendix* and therefore more experiments were conducted with the *Sauromatum* appendix. Another advantage of the *Sauromatum* appendix is that the inflorescence can develop from a corm without any soil.

### Induction of a thermogenic response

Pre D-day appendices of the *Sauromatum* and *Arum* inflorescences were cut transversely into equal length sections and immediately placed either in inducer solutions with different final concentration or in a control solution (without the inducer) in a 96-well plate in an environmental chamber (SANYO) under constant light and temperature. Each set of experiments was performed with sections from one appendix. The *Sauromatum* appendix was cut into sections of 2–3 cm in length and *Arum* appendix was cut into sections of ~0.5 cm in length. The sections were placed in an appropriate solution containing SA, ASA, or 2,6-DHBA in 2.5 mM HEPES buffer, pH 7.0. The appendix sections were in the solution throughout the experimental period. Control appendix sections were treated in the same manner but without the inducers. The sectioning and placing of the sections in various solutions were completed in ~1 min for the *Sauromatum* appendix and ~1 h for the *Arum* appendix. Data collection started as soon as the appendix sections were placed in the appropriate solution.

### Temperature measurement

T-type thermocouple probes 0.5 mm in diameter (copper/constantan, TMTSS-020G, Omega Engineering, Stamford, CT) were inserted into the appendix sections and the temperature was recorded every 2 min with a data logger (Omega Engineering). The temperature sensitivity of T-type thermocouples is liner at physiological temperatures. There were 720 consecutive readings per 24 h. The effect of a treatment on the temperature rise was calculated as the difference in temperature between treated and untreated sections. Temperature rate was defined as Δ°C/min and was calculated by subtracting consecutive readings and dividing by time (2 min). The temperature rate data was smoothed using Excel’s Moving Average function.

## Results

### Temperature wave induced by ASA and 2,6-DHBA in the Sauromatum appendix

**Figure 1:**
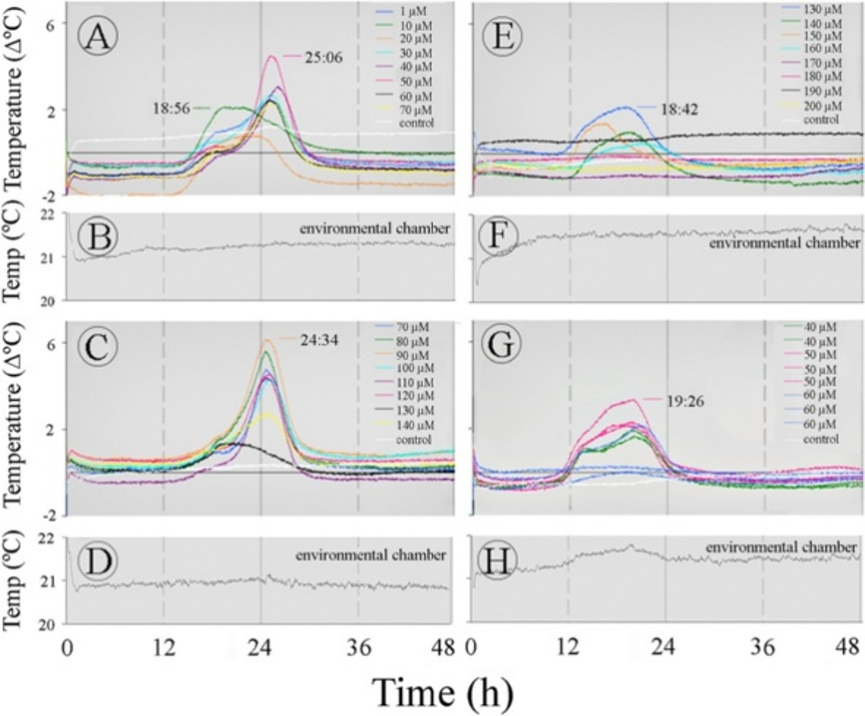
**Temperature profiles of the** ***Sauromatum*** **appendix in the presence of ASA.** Each set of the treatments in **A**, **C**, **E**, and **G** was performed with one pre D-day appendix. The gray line in **B**, **D**, **F**, and **H** shows the temperature in the environmental chamber for **A**, **C**, **E**, and **G**, respectively. The white line represents the temperature of the control (treated with buffer only) minus the temperature in the environmental chamber. Each line color represents one concentration of inducer and is a subtraction of the control from the treatment. A negative or positive temperature value of the control means that its temperature was either lower or higher than the temperature in the environmental chamber.

The profile of the temperature of sections from the *Sauromatum* appendix was studied with 10-μM intervals of ASA, from 0 to 200 μM (Figure 1). A slow rising temperature was observed after a ~13-h period of a constant baseline temperature. The temperature peak fluctuated back and forth with time from ~19 to 25 h at various concentrations of ASA (Figure 1A, C and E). The temperature amplitude fluctuated as well, from 0 to 6°C above environmental chamber temperature. The peak at ~19 h was evident at 1–10 μM and 140–200 μM ASA and the peak at ~25 h was evident at 30–120 μM ASA. ASA was present in the inducer solution throughout the treatment and it is unlikely that the appearance of two maxima at different times is a result of changes in ASA concentrations in the appendix tissue. It also appeared that when the intensity of the major peak at ~25 h was low the peak at 19 h was high (e.g., 10 μM versus 50 μM ASA in A). It implies an inverse correlation between the two successive temperature maxima.

**Figure 2:**
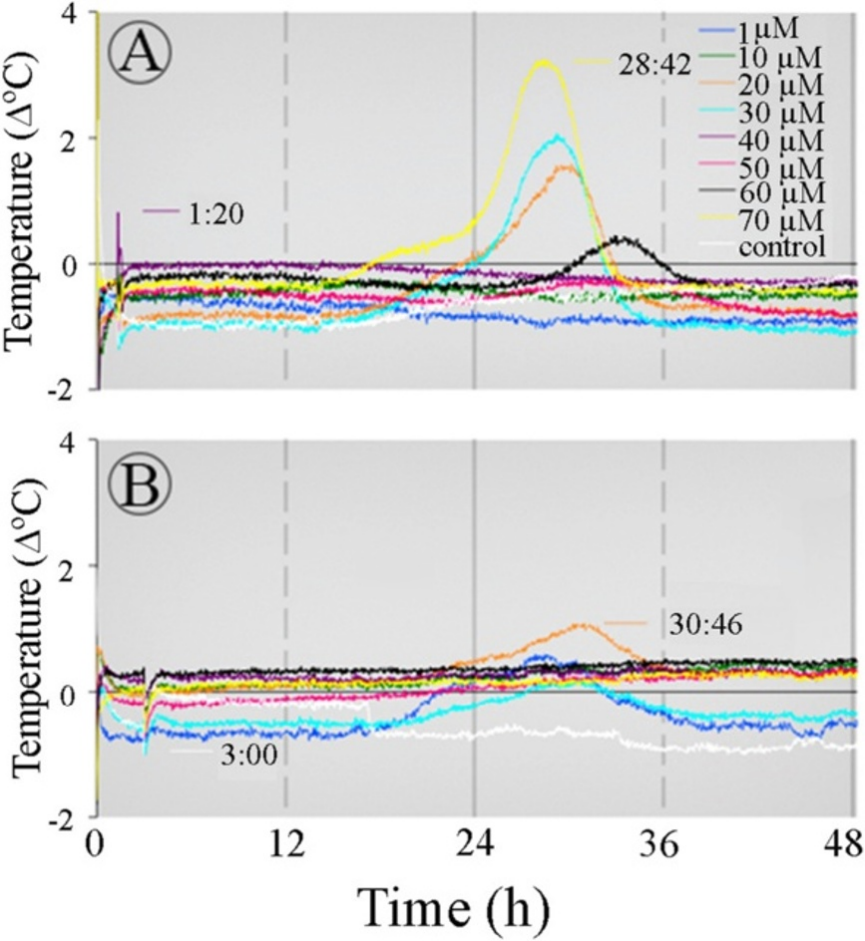
**Induction of a time keeping mechanism by ASA in the** ***Sauromatum*** **appendix.** Sections of one pre D-day appendix were placed in the control solution (treated with buffer only) for 1:20 h **(A)** and sections of a second pre D-day appendix were placed in the control solution for 3 h **(B)**. Subsequently, ASA was added at various concentrations. Opening of the door of the environmental chamber in order to add ASA to the control solution caused a temperature spike at 1:20 h and 3 h from the beginning of the experiment. Environmental chamber temperature was ~21°C. The legends are identical for **A** and **B**. Temperature profiles are a subtraction of the control from a treatment.

To separate ASA induction from sectioning effect, ASA was added only after the sections were present in the buffer solution for 1:20 h or 3 h (Figure 2A-B). When sections were first placed in the buffer solution for ~1 h and later in the ASA solution, the temperature reached a maximum at ~27 h from the beginning of the ASA treatment (Figure 2A). The time to reach a maximum was similar when ASA was added after 3 h. These results indicate that ASA affects an endogenous time keeping mechanism that is involved in the process that leads to the temperature rise. The second maximum at 20 h from the beginning of the treatment could barely be detected in this set of treatments.

The variations in temperature induction by ASA are further illustrated in Figure 1G. When sections of one appendix were treated with ASA in duplicate or triplicate only the peak at ~19 h was detected, and the temperature did not rise at all in two out of three sections at 60 μM ASA. The variability in temperature did not seem to be a result of heat loss because the thermocouples were inserted into the tissue and they recorded the section temperature where heat loss to the environment is small. These variations were observed in two D-3 appendices treated with SA, one D-2 appendix treated with ASA, and one D-1 appendix treated with 2,6-DHBA. Additionally, variations in temperature were observed in six appendices treated with SA or ASA and one appendix treated with 2,6-DHBA at pre D-day stage. Consequently, each set of treatments described in the results was carried out with one appendix.

**Figure 3:**
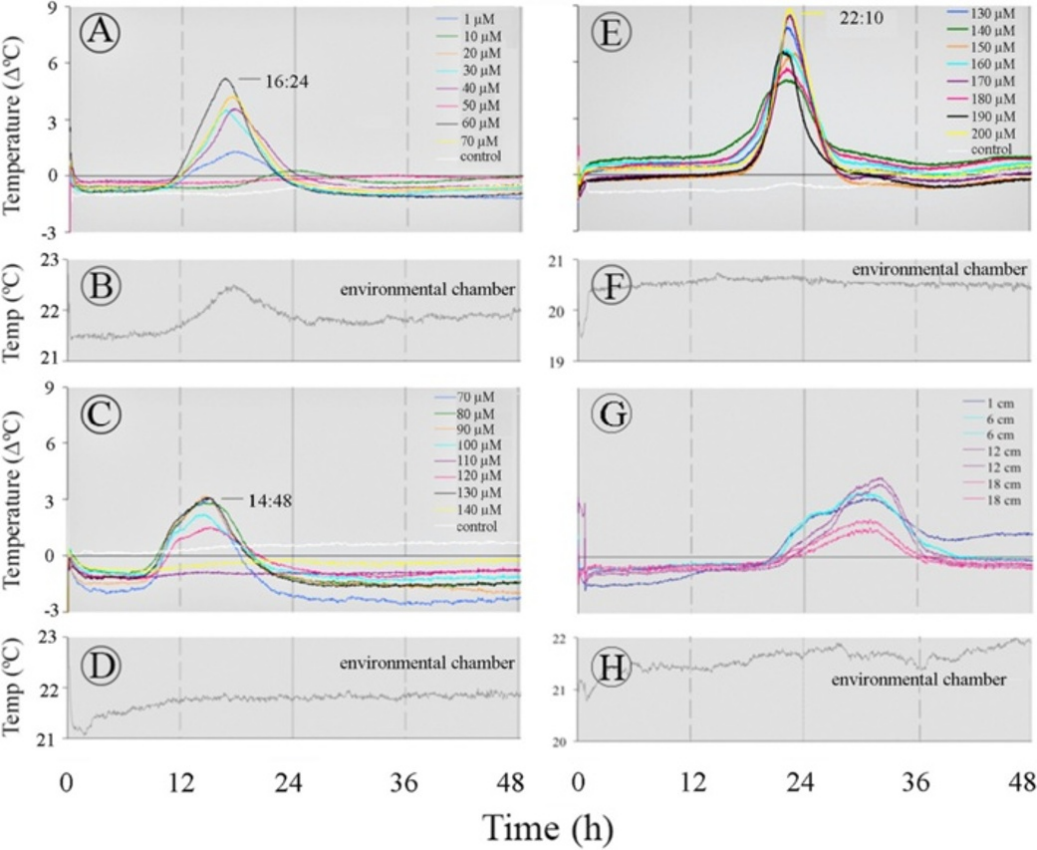
**Temperature profiles of the** ***Sauromatum*** **appendix in the presence of 2,6-DHBA.** Each set of treatments of in **A**, **C**, and **E** was performed with one pre D-day appendix. The gray line in **B**, **D**, **F**, and **H** shows the environmental chamber temperature for **A**, **C**, **E**, and **G** respectively. The temperature along an appendix of an intact inflorescence in shown in **(G)**. Each line color **(G)** represents a distance from the appendix base and is a subtraction of the environmental chamber temperature from the appendix temperature. Temperature profiles in **A**, **C**, and **E** are a subtraction of the control from a treatment.

The appearance of two successive temperature maxima was also observed with 2,6-DHBA (Figure 3). A temperature maximum appeared at ~15 h after the inducer application and a second maximum appeared at 22 h. Even in the most responsive appendix sections (Figure 3E) in which the temperature rose 9°C above the control, the temperature amplitude fluctuated from 5 to 9°C. The changes in the timing of temperature maximum were not affected by the inducer concentration. At higher concentrations of 2,6-DHBA the temperature maximum (Figure 3E) appeared later than that at lower concentration (Figure 3A).

In order to account for the temperature differences seen among sections of the appendix, the temperature of an appendix of an intact inflorescence was also recorded (Figure 3G). It seems that the rise in temperature was lower at the tip of the appendix (18 cm from the base) than in the middle part (6–12 cm from the base) and the base. The appearance of two successive temperature maxima was also observed with the intact appendix. The two temperature maxima were seen at the base and the middle part of the appendix (1–6 cm). The temperature was also recorded at two parallel points along the vertical axis of the appendix at 6 and 12 cm from the base. No difference in temperature was observed at these vertical points indicating that the heat is distributed uniformly over the vertical axis. The moderate temperature gradient along the horizontal axis of the appendix can be explained by the difference in the appendix diameter. The base of the appendix is wider than its tip and therefore, it may better retain heat than its tip. Consequently, the tip temperature can be lower than the base temperature.

### Temperature wave induced by SA, ASA and 2,6-DHBA in the Arum appendix

**Figure 4:**
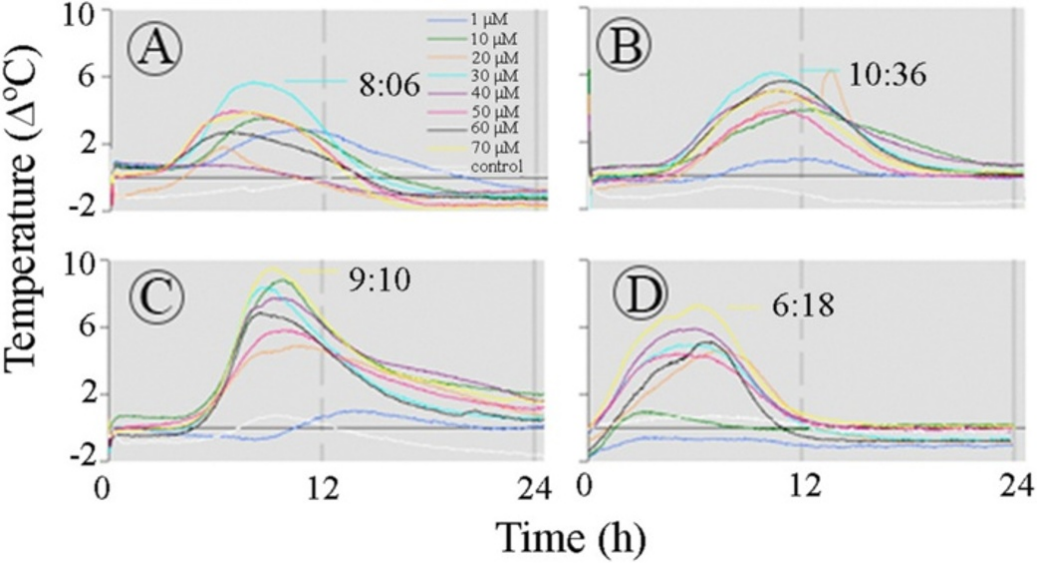
**Temperature profiles of the** ***Arum*** **appendix in the presence of thermogenic inducers.** The temperature of sections of the *Arum* appendix were recorded over 24 h at various concentrations of inducers. Sections of one appendix were placed in the appropriate solutions and immediately connected to thermocouples. Each set of treatments with SA **(A-B)**, ASA **(C)**, and 2,6-DHBA **(D)** was performed with one pre D-day appendix. Environmental chamber temperature was ~21°C. The legends are identical for **A**-**D**.

The temperature profile of sections from the pre D-day *Arum* appendices was studied with 10-μM intervals, from 0 to 70 μM (Figure 4). Variability in the temperature rise was also evident in the profile of temperature obtained from two pre D-day appendices treated with SA (A-B), ASA (C), and 2,6-DHBA (D). The temperature maximum was between 6 to 11 h from the inducer application and the temperature amplitude fluctuated from 0 to 10°C. A temperature peak around 24 h from the inducer application that was evident in the *Sauromatum* appendix was not detected in the *Arum* appendix.

### Two successive temperature rate maxima

**Figure 5:**
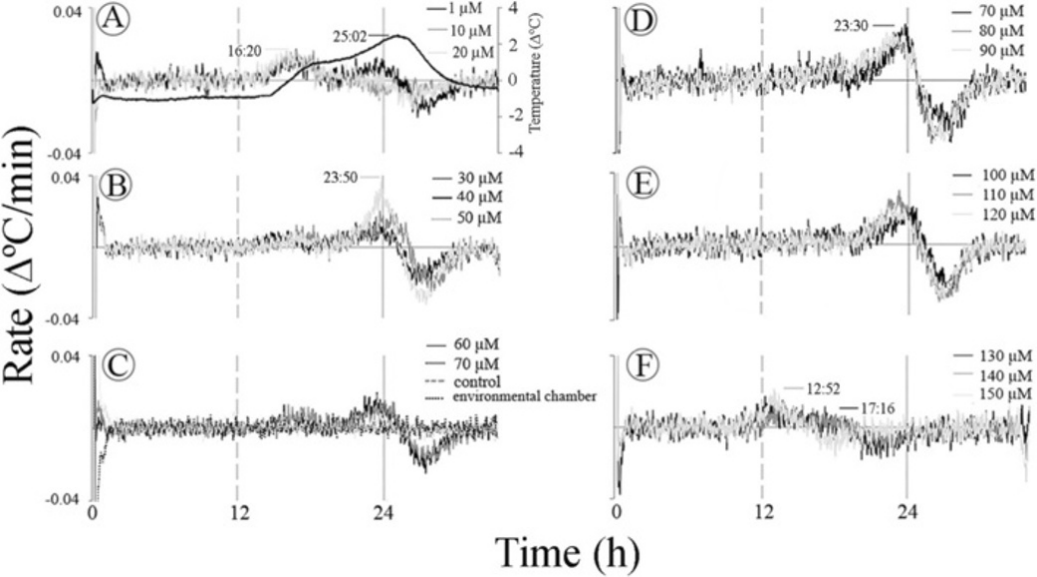
**Rate of rising temperature in the** ***Sauromatum*** **appendix in the presence of ASA.** Temperature values from Figure 1 were used to calculate temperature rate at various concentrations of ASA **(A-F)**. Rate was expressed as a the change in temperature per min. Dashed black line in **(C)** is the temperature rate in the environmental chamber and the gray dashed line is the control. The black thick line **(A)** is the temperature in the presence of 1 μM ASA superimposed on the temperature rate.

Changes in temperature rise rate per min were calculated in an attempt to understand the dynamics of the temperature rise *in the Sauromatum appendix*. This type of calculation allows an accurate comparison between different temperature profiles. Figure 5A-F shows the temperature rates in the presence of ASA. Immediately after sectioning, the temperature rates drastically changed and it took about 1 h to reach a steady state baseline. Subsequently, the temperature rate reached two successive maxima at ~17 h and 24 h (A, B, and D). Two temperature maxima were also detected in appendices of intact inflorescences. One plausible explanation for the appearance of two distinct maxima is based on the assumption that the mitochondria are the only source of heat and thus the different times of rate maxima represent interplay between two heat fluxes in the mitochondria. One is presumably from the AOX activity and the other is from the Cyt pathway. The rate of ~0.04 Δ°C/min may represent a single source of heat. Another plausible explanation is that each rate maximum represents a distinct source of heat generating system, and only one of them is from the mitochondria.

Another characteristic of the the temperature maxima was that it always followed the rate maxima by ~1–3 h. When the temperature rate reached its maximum it slowed down and crossed the x-axis at the temperature maximum. The rate values became negative when the temperature decreased and when the rate values returned to zero the temperature returned to baseline. The time lag between the temperature peak and rate maximum is only shown for 1 μM ASA treatment (Figure 5A). This lag in time was observed at all concentrations with the three inducers. These results suggest the presence of a control mechanism over the temperature maximum. The temperature rate of the control and environmental chamber had a slope of zero that did not change over time (C). It also appears that when the rate peak at the ~23 h was high (D and E) the rate peak at ~17 h was low, and when the peak at ~17 was high (A) the peak at ~23 was relatively low. In (F) a rate maximum at 13 h was detected and it could be associated with the maximum at 17 h.

In *Arum* appendix one rate maximum was detected when appendix sections were treated with 1-70 μM ASA and SA. The maximum rate was 0.07 Δ°C/min with ASA treatment and 0.03 Δ°C/min with SA treatment, and it occurred at ~7 h from the beginning of the treatment.

**Figure 6:**
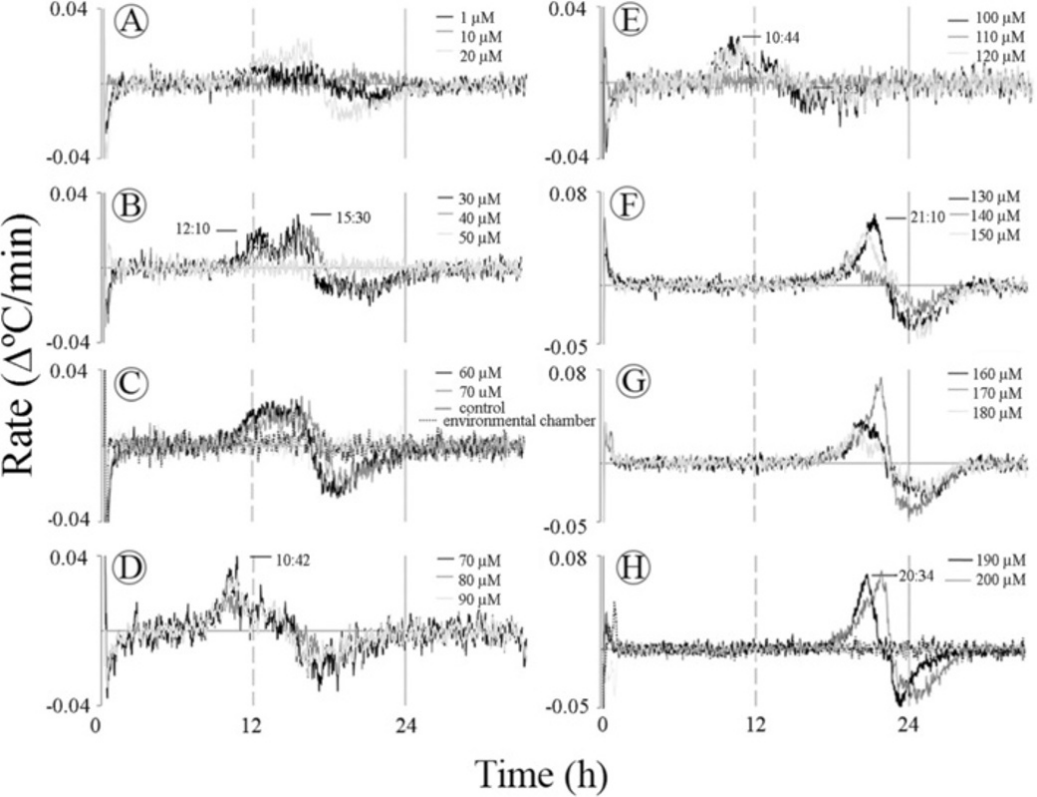
**Rate of rising temperature in the** ***Sauromatum*** **appendix in the presence of 2,6-DHBA.** Temperature values from Figure 3 were used to caluculate temperature rate at various concentrations of 2,6-DHBA **(A-H)**. Rate was expressed as in Figure 5.

Two major rates of temperature rise were also evident when the appendix sections were treated with 2,6-DHBA (Figure 6). At 30–50 μM (B) two successive rate maxima appeared at ~12 and 15 h from the beginning of the treatments. At 100–120 μM (E) the rate maximum at ~12 h was higher than that at ~15 h. At 130 to 200 μM (F-H) a new rate maximum appeared at ~20 h. The high rate of 0.07 Δ°C/min may suggest that either the heat-generating source is not saturated at 0.03 Δ°C/min or, that this rate is the sum of two separate but synchronized and overlapping rate maxima.

**Figure 7:**
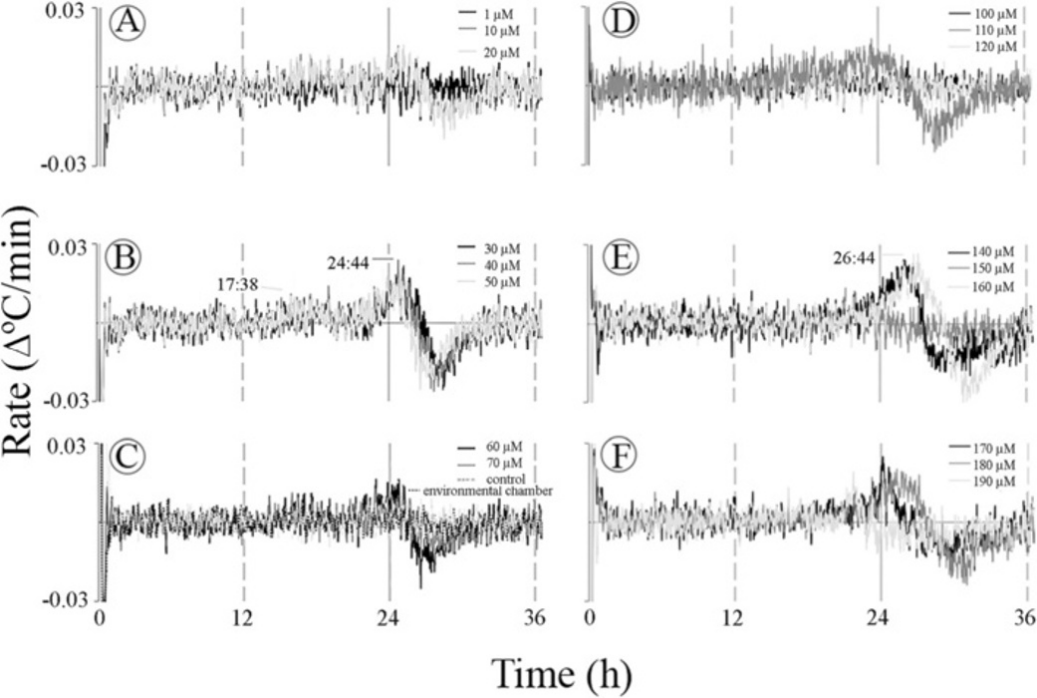
**Rate of rising temperature in the** ***Sauromatum*** **appendix in the presence of SA.** Temperature values were used to calculate temperature rate at various concentrations of SA. **(A-F)**. Rate was expressed as in Figure 5.

In the case of SA, two rate maxima at ~17 h (Figure 7B) and 25 h (B, E, and F) were also detected at various concentrations. The temperature maximum at 24 h was the major peak at increasing concentrations of SA, from 1 to 190 μM (A-F). However, it also appears that the two rate maxima exhibited opposing states. When one rate was going up the other one was suppressed. This may suggest the existence of a feedback mechanism between two major heat-generating sources.

The appearance of two rat maxima is not a result of the variations within an appendix. In many concentrations the two maxima appeared together at a single concentration of the inducer. For example, in Figure 4A-C two sequential maxima were detected at the same concentration of ASA. The two rate maxima were also detected when sections were treated with 2,6-DHBA (Figure 6A and E) and with SA (Figure 7A-B).

### Temperature oscillation

**Figure 8:**
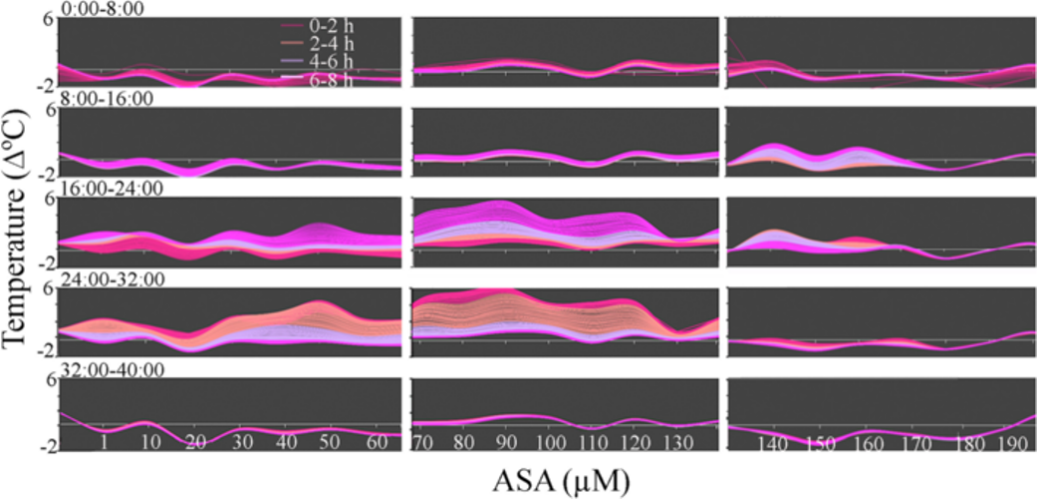
**Temperature oscillation in the** ***Sauromatum*** **appendix as a function of time and ASA concentration.** Forty-eight h of treatment with ASA were divided into 4 groups of 8 h. Each group was further subdivided into 4 groups of 2 h. Each subdivision is represented by a different line color that depicts the temperature in a particular time of ASA treatment. Temperature data from 3 pre D-day appendices (one appendix treated with 1-70 μM, second appendix with 70-140 μM, and third appendix with 140-210 μM) shown in Figure 1 were used to produce this figure.

A moderate temperature gradient was observed along the appendix and it raises the question whether a temperature gradient exists because of changes in SA concentration in the appendix tissue.x. Figure 8 provides a quantitatively information on how changes in the inducer concentrations may cause different oscillatory patterns over time in the *Sauromatum* appendix. ASA triggered a temperature wave in which temperatures went up and down as ASA concentrations increased. It seems that the overall temperature profile stayed the same from the beginning of the treatment and only the amplitude of the temperature wave varied over time. This kind of analysis can provide a temperature profile across various concentrations of an inducer at a particular time.

### Temperature set-point

**Figure 9:**
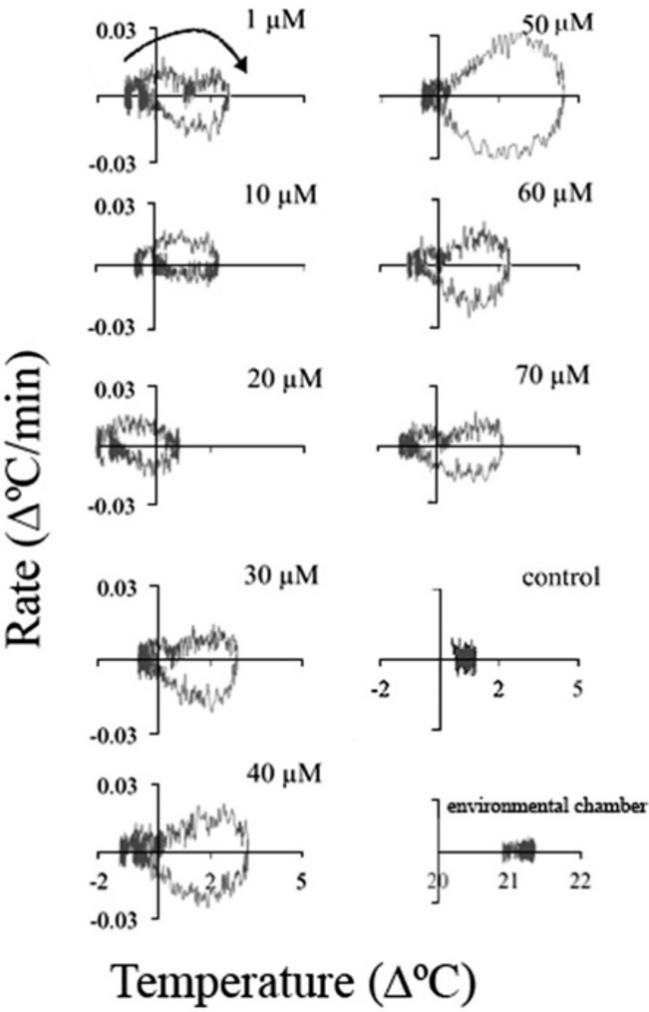
**Temperature set-point in the** ***Sauromatum*** **appendix in the presence of ASA.** Temperature rates from Figure 5 were plotted against temperature values from Figure 1. Concentration of ASA is indicated on the right side of each plot. Arrow points to the direction of temperature rise.

Another method of obtaining insights on the nature of temperature rise is by plotting the rate of temperature rise against the temperature (Figure 9). Again, two successive temperature waves were clearly observed at 1, 30, 60, and 70 μM ASA. It seems that there was a pause in the temperature rise between the two waves. The temperature rate and temperature value of the control (an untreated appendix section) and the environmental chamber remained unchanged during the 48-h treatment. All the appendix sections treated with ASA were colder than the control at the beginning of the treatment. At some ASA concentrations (1, 10, 20, and 40 μM) the temperature after 48 h did not returned to the initial level and a new steady level of 0.1-0.5°C higher than the initial temperature was reached. It seems that when the second temperature wave with a maximum at ~24 h was absent or low the final temperature was higher than the initial one. The increase in the baseline temperature was small but it may indicate changes in energy metabolism. These changes in baseline temperature also indicate the presence of a thermoregulatory mechanism in the *Sauromatum* appendix and its modulation by ASA.

**Figure 10:**
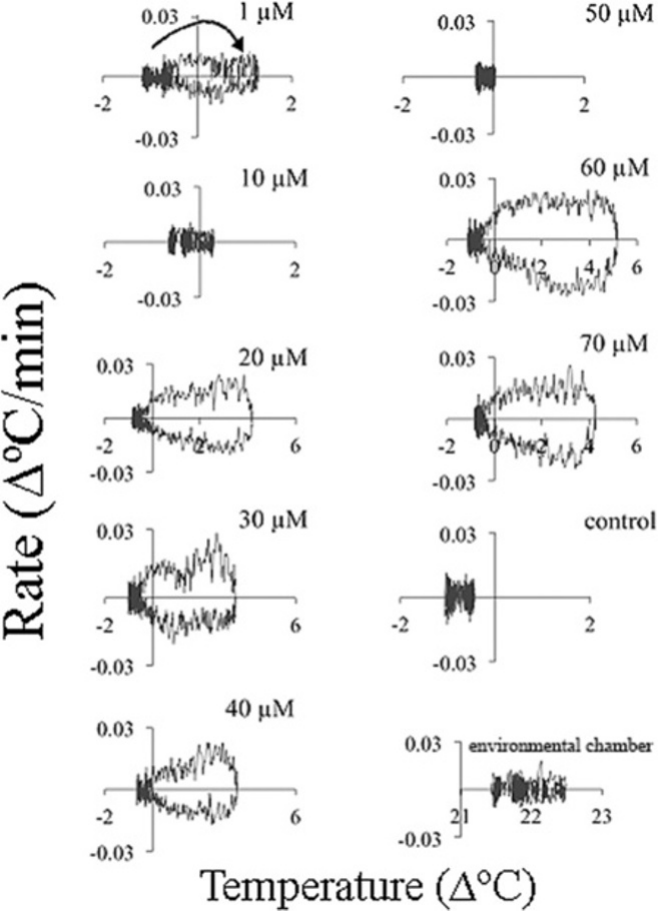
**Temperature set-point in the** ***Sauromatum*** **appendix in the presence of 2,6-DHBA.** The temperature rates from Figure 6 were plotted against temperature values from Figure 3. Concentration of 2,6-DHBA is indicated on the right side of each plot. Arrow points to the direction of temperature rise.

Treatment with 2,6-DHBA also displayed two temperature waves that are clearly seen at 30 and 40 μM (Figure 10). However, no other changes in the temperature set-point were detected at other concentrations, except that at 1 μM the final temperature was lower than the initial one. The control temperature was lower than the environmental chamber temperature.

**Figure 11:**
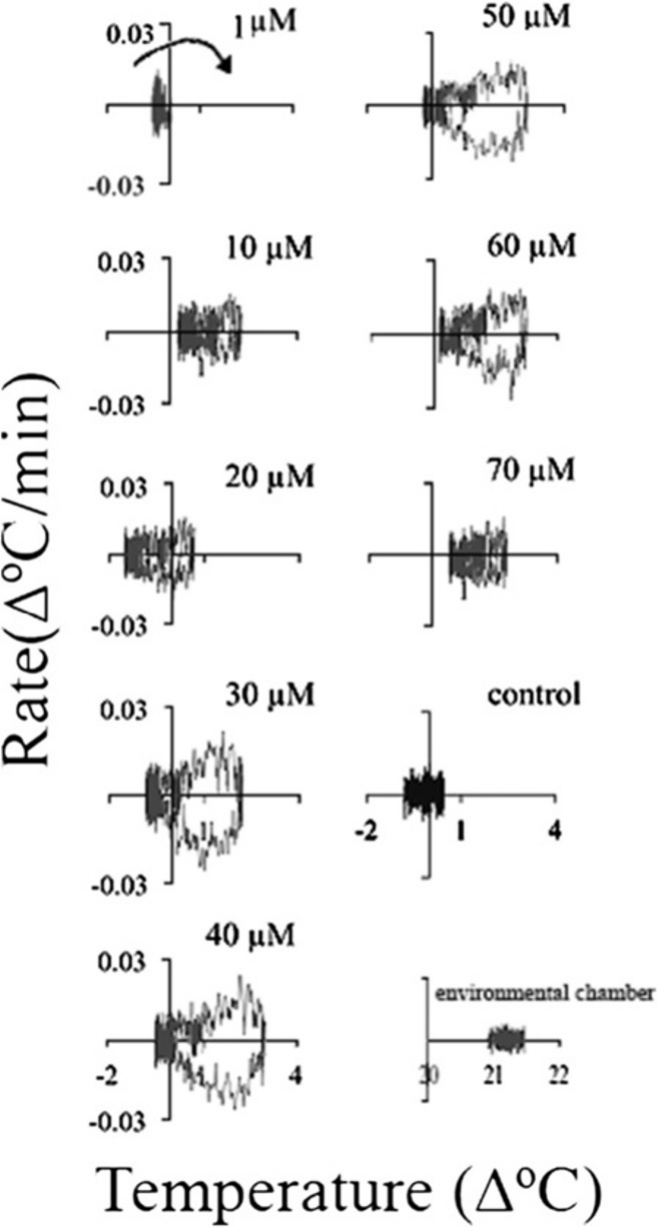
**Temperature set-point in sections of the** ***Sauromatum*** **appendix in the presence of SA.** The temperature rates from Figure 7 were plotted against temperature value. Concentration of SA is indicated on the right side of each plot. Arrow points to the direction of temperature rise.

Two successive temperature waves were also detected in the presence of SA from 20–60 μM (Figure 11). The final temperature was kept either unchanged or slightly lower (50 and 60 μM).

### Effect of KCN and SHAM on temperature rise

**Figure 12:**
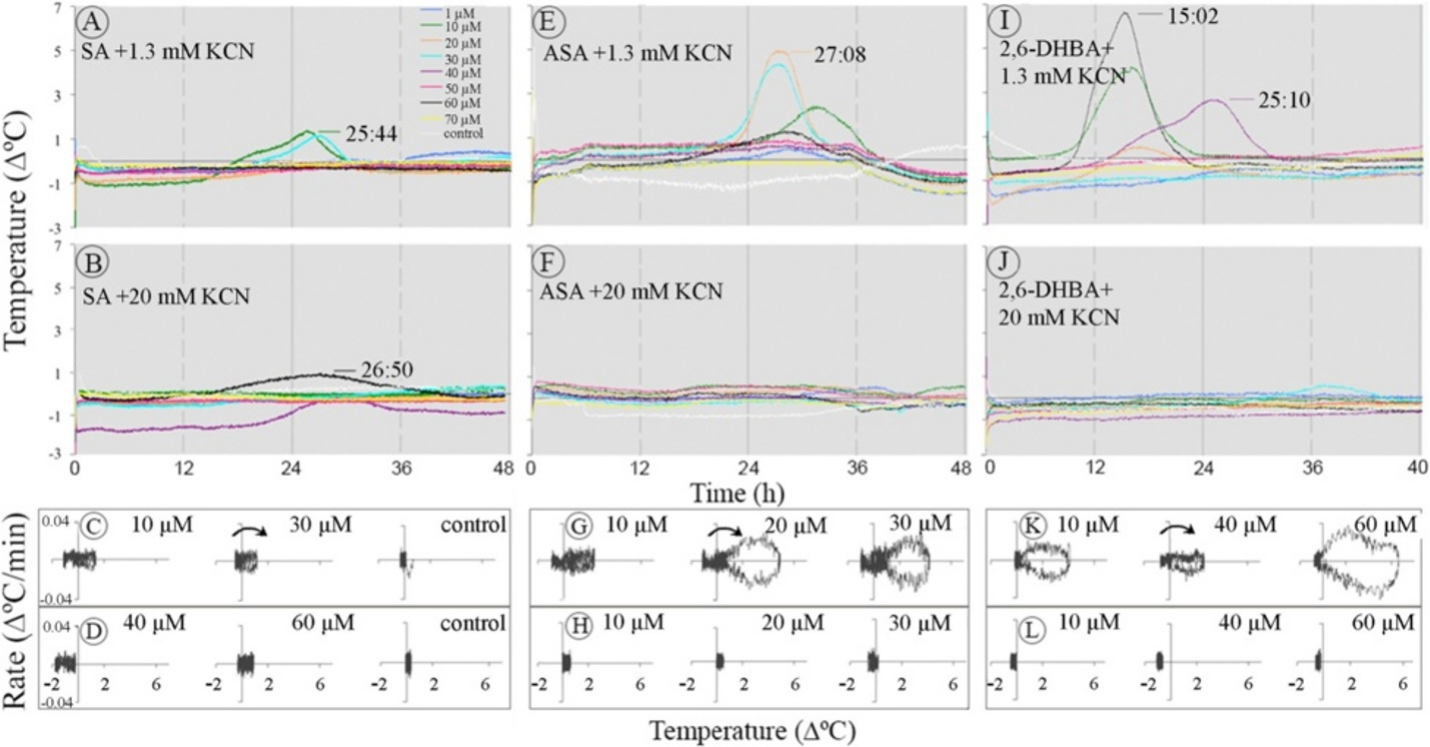
**Effect of KCN and thermogenic inducers on temperature rise in the** ***Sauromatum*** **appendix.** Each set of treatments was carried out with one pre D-day appendix. KCN was added to various concentrations of inducer. The plots of temperature rate against temperature value **(C**, **D**, **G**, **H**, **K, and** **L)** were derived from **A**, **B**, **E**, **F**, **I**, and **J**, respectively. The legends are identical for **A**, **B**, **E**, **F**, **I**, **J**. Control sections were treated only with the buffer solution containing KCN at the appropriate concentration. Environmental chamber temperature was ~21°C. The legends are identical for **A, B, E, F, I, and J**. Concentration of SA **(C** **and D)**, ASA **(G and** **H)**, and 2,6-DHBA **(K and** **L)** is indicated on the right side of each plot.

Application of KCN at final concentration of 1.3 mM decreased the temperature rise induced by SA, ASA, and 2,6-DHBA in sections from *Sauromatum* appendices at pre D-day (Figure 12A, E, and I). When the temperature rate was plotted against the temperature value only one temperature peak at 26–27 h was detected in sections treated with 1.3 mM KCN and ASA (G). Two temperature waves were detected in the presence of 2,6-DHBA at 15 h and 25 h (K). KCN at 20 mM prevented the rise in temperature, most likely by blocking the operation of the Cyt pathway (Figure 12B, D, F, H, J, and L). There also appeared to be no differences in the response to 20 mM KCN in the presence of ASA in thick (2–3 cm) or thin (0.5 cm) sections.

This finding is hard to reconcile with the concept that heat is generated by AOX that is resistance to KCN. One explanation for this effect is that the operation of the Cyt pathway prior to the temperature rise is required for some unknown functions. Another explanation is the activity of UCPs to generate heat is decreased because of the inhibition of COX by KCN.

**Figure 13:**
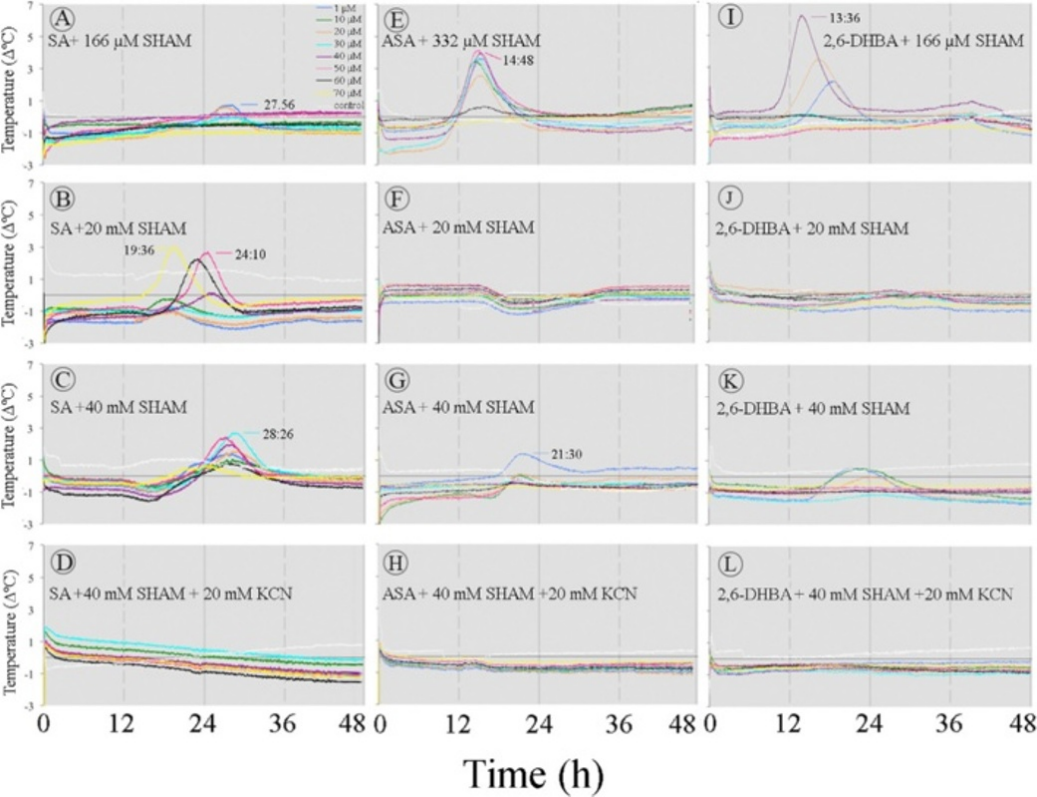
**Effect of SHAM and thermogenic inducers on temperature rise in the** ***Sauromatum*** **appendix.** SHAM was added to the inducer solution at various concentrations. Each set of treatments with SHAM was carried out with one pre D-day appendix. The legends are identical for **A**-**L**. Control sections were treated only with the buffer solution containing SHAM or SHAM and KCN combination at the appropriate concentrations. Environmental chamber temperature was ~21°C.

Application of SHAM, an AOX inhibitor, significantly blocked the temperature rise induced by SA, but it did not block it completely (Figure 13A-C). Treatments with ASA (E-F) and 2,6-DHBA (I-K) plus SHAM produced similar decreases in temperature rise. Even at 40 mM SHAM, the temperature slightly rose. This residual temperature rise was sensitive to KCN because it was completely blocked when the application of KCN and SHAM was combined (Figure 13D, H, and L).One explanation for the residual temperature rise is that SHAM does not readily cross the cell wall and the plasma membrane and therefore the inhibition was incomplete.

### Effect of DNP on temperature rise

**Figure 14:**
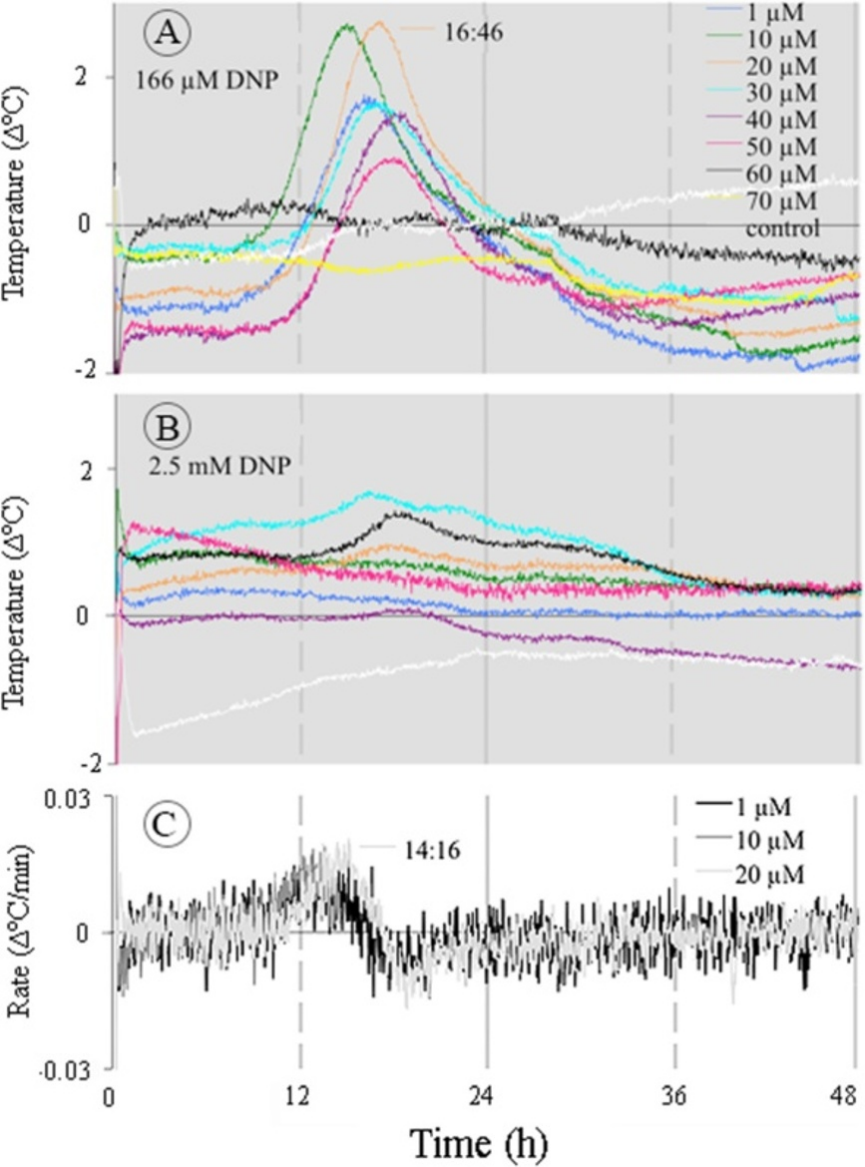
**Effect of DNP and ASA on temperature rise in the** ***Sauromatum*** **appendix.** DNP was added to the buffer solution containing inducers at various concentrations. Each set of treatments with DNP **(A and** **B)** was carried out with one pre D-day appendix. Temperature rates were calculated from temperature values in **A** and plotted against the temperature values **(C)**. The legends are identical for **A**-**B**. Control sections were treated only with the buffer solution containing DNP at the appropriate concentrations. Environmental chamber temperature was ~21°C.

To test the possibility that the some of the heat was generated by UCPs activity, sections of the *Sauromatum* appendix were treated with DNP, an uncoupler agent, in the present of ASA (Figure 14A-C). If UCPs were active, then the addition of DNP would make no difference in the temperature rise because the proton gradient was already dissipated. The temperature would rise in case the proton gradient was only partly dissipated by UCPs. However, if the AOX were active and the Cyt pathway saturated, then DNP would facilitate the electron flow via Cyt pathway and the temperature would decrease. Indeed, DNP at 2.5 mM decreased the rise in temperature to ~1°C in the presence of ASA. DNP at this concentration most likely uncouples mitochondrial proton transport from phosphorylation. The interpretation of this result is that UCPs are most likely not involved in the rising temperature and the Cyt pathway activity that generates a membrane potential exerts a control over the temperature rise. It is also interesting that only the early temperature maximum (~15 h) was detected at 166 μM DNP (C). It appears that this early maximum is more sensitive to KCN and less sensitive to DNP than the temperature maximum at ~24 h.

## Discussion

### Two temperature waves

Two temperature maxima were observed in sections and intact appendices of the *S. guttatum* inflorescence. The temperature maxima seem to be coupled and the coupling is partially controlled by the concentrations of the thermogenic inducers. These data raise the question whether the two temperature waves represent one heat source, the mitochondria or, two different sources of heat. One source can be AOX that is blocked by SHAM and the other source can be a cyanide-sensitive activity, presumably, the Cyt pathway and/or another activity that is affected by KCN. It is conceivable that in the presence of an inducer coupling between the two sources occurs and heat is generated. The coupling strength between the two sources can vary and each source can also operate individually. For example, 2,6-DHBA may trigger the two sources separately (Figure 3C versus E) whereas ASA (Figure 1) and SA may trigger them simultaneously. If this hypothesis is correct than the time between the two temperature waves represents the time of the feedback between the two temperature waves.

In our previous publications, we have described the isolation of a novel 34.1 kDa NAD(P) reductase like protein (RL) from the thermogenic appendix of the *Sauromatum* inflorescence (Skubatz and Howald [2013]). We also have shown that RL is present in two global conformations. An extended conformation (state A) that is only present at pre D-day when SA is undetectable, and a compact conformation (state B) that is present on D-day, when SA level is high. The protein was present in state B during the rise in temperature in thermogenic organs of several other members of the Araceae family and in thermogenic plants of other plant families. In *Arabidopsis* leaves, a non-thermogenic plant, it was present in state A. Furthermore, addition of SA at pM concentration to the purified protein solution induced a reversible volume phase transition every 4–5 min for at least 30 min (Skubatz et al. [2013]). These changes in the protein conformation may result in variation in the response to the inducers. It is also conceivable that this protein induces two systems to generate heat by conformational changes.

### Inhibition of temperature rise

The fact that KCN blocked the rise in temperature is surprising. The 24-h incubation of the appendix sections with KCN should not affect AOX activity. In Cocklebur seeds (*Xanthium strumarium*) pretreatment with 30 mM KCN for 16 h did not abolish AOX activity (Esashi et al. [1981]). It is plausible that KCN binds to COX and to another protein(s) involved in the temperature rise. A number of enzymes are inhibited by KCN, for example, catalase (Wolfe et al. [1957]) and malic dehydrogenase (Zołnierowicz et al. [1988]).

The effect of DNP on the rise in temperature is also puzzling. If UCPs were active on D-day then DNP should not block the temperature rise because the mitochondrial proton gradient had been partially dissipated. Uncoupling of mitochondrial oxidative phosphorylation could have a negative impact when ATP production is needed prior to D-day. On D-day, the ATP/ADP ratio is low ~0.9 maybe because of a high ATP turnover rate (Skubatz et al. [1992]). In a previous study, when DNP was applied to sections of the *Sauromatum* appendix at a micromolar range no significant effect on the respiration rate on D-1 and D-Day was detected, but the respiration rate increased when it was applied post D-Day (Hess and Meeuse [1967]).

## Conclusions

By reexamination of temperature data, this study has demonstrated the presence of two successive temperature rates and the capability of the appendix tissue to maintain and change its initial temperature. These *in vivo* results do not correspond to the cyanide-insensitive respiration of isolated mitochondria. They may indicate the involvement of cellular components not present in isolated mitochondria.

## Authors’ original submitted files for images

Below are the links to the authors’ original submitted files for images. Authors’ original file for figure 1 Authors’ original file for figure 2 Authors’ original file for figure 3 Authors’ original file for figure 4 Authors’ original file for figure 5 Authors’ original file for figure 6 Authors’ original file for figure 7 Authors’ original file for figure 8 Authors’ original file for figure 9 Authors’ original file for figure 10 Authors’ original file for figure 11 Authors’ original file for figure 12 Authors’ original file for figure 13 Authors’ original file for figure 14 Authors’ original file for figure 15

## Abbreviations

ASA: Aspirin, acetylsalicylic acid
AOX: Alternative oxidase
COX: Cytochrome oxidase
Cyt: Cytochrome
D-day: The day of heat-production
2: 6-DHBA: 2,6 Dihydroxybenzoic acid
DMSO: Dimethyl sulfoxide
DNP: 2,4-Dintrophenol
KCN: Potassium cyanide
Pre D-day: Three to 1 days prior to D-day
SA: Salicylic acid
SHAM: Salicylhydroxamic acid
UCPs: Uncoupling proteins
