# Supplementary figures and images for: Thermoregulation in the appendix of the Sauromatum guttatum (Schott) inflorescence
Source: Bot Stud. 2014 Sep 26;55:68. doi: 10.1186/s40529-014-0068-0 (PMC5430309; doi:10.1186/s40529-014-0068-0)

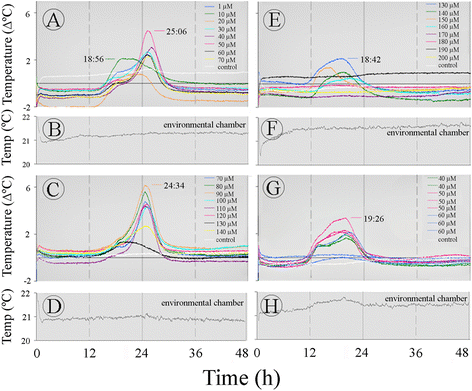

Supplement: Supplementary file 1 — Authors’ original file for figure 1 [file 40529_2014_9068_MOESM1_ESM.gif]

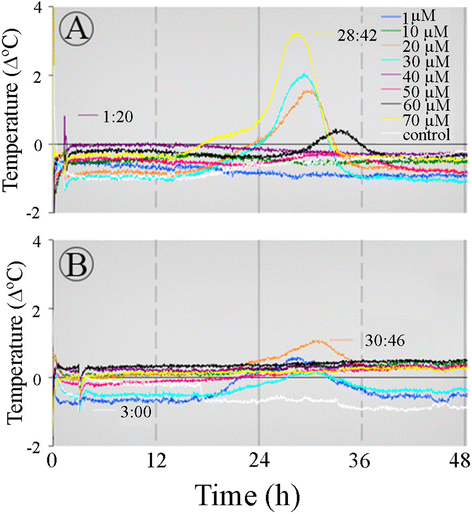

Supplement: Supplementary file 2 — Authors’ original file for figure 2 [file 40529_2014_9068_MOESM2_ESM.gif]

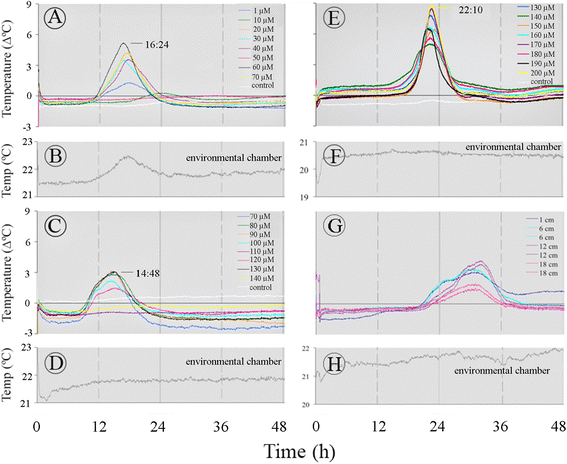

Supplement: Supplementary file 3 — Authors’ original file for figure 3 [file 40529_2014_9068_MOESM3_ESM.gif]

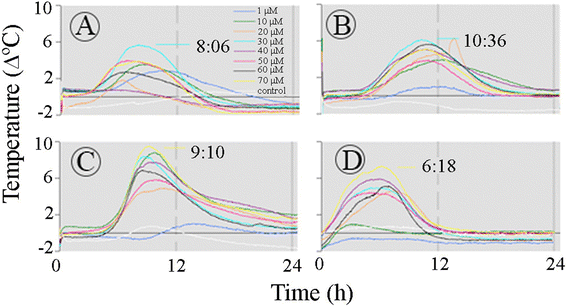

Supplement: Supplementary file 4 — Authors’ original file for figure 4 [file 40529_2014_9068_MOESM4_ESM.gif]

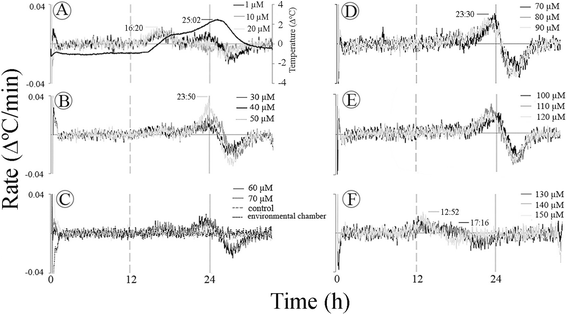

Supplement: Supplementary file 5 — Authors’ original file for figure 5 [file 40529_2014_9068_MOESM5_ESM.gif]

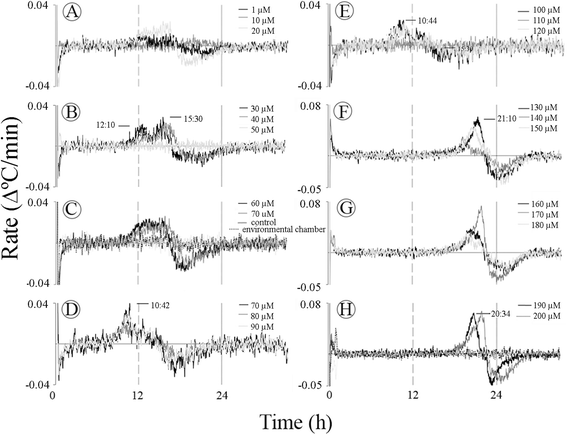

Supplement: Supplementary file 6 — Authors’ original file for figure 6 [file 40529_2014_9068_MOESM6_ESM.gif]

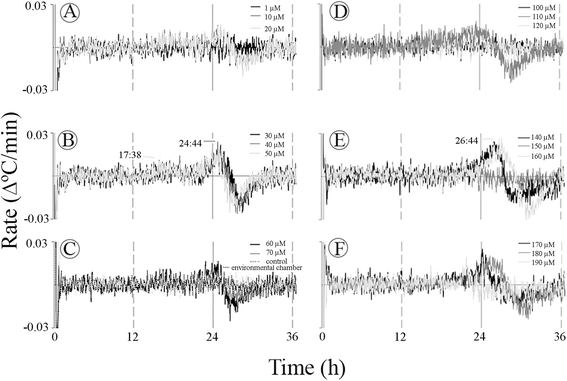

Supplement: Supplementary file 7 — Authors’ original file for figure 7 [file 40529_2014_9068_MOESM7_ESM.gif]

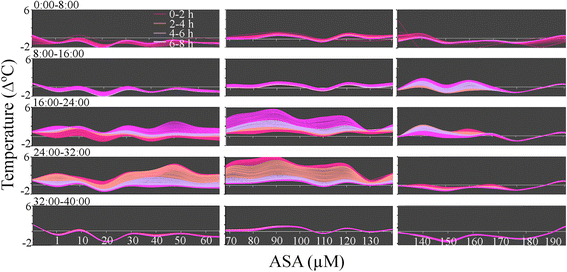

Supplement: Supplementary file 8 — Authors’ original file for figure 8 [file 40529_2014_9068_MOESM8_ESM.gif]

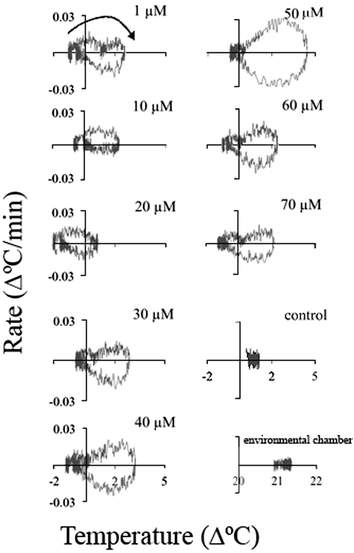

Supplement: Supplementary file 9 — Authors’ original file for figure 9 [file 40529_2014_9068_MOESM9_ESM.gif]

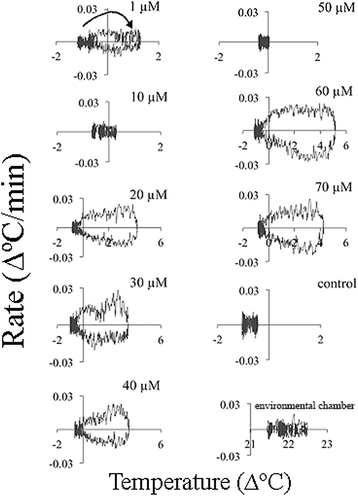

Supplement: Supplementary file 10 — Authors’ original file for figure 10 [file 40529_2014_9068_MOESM10_ESM.gif]

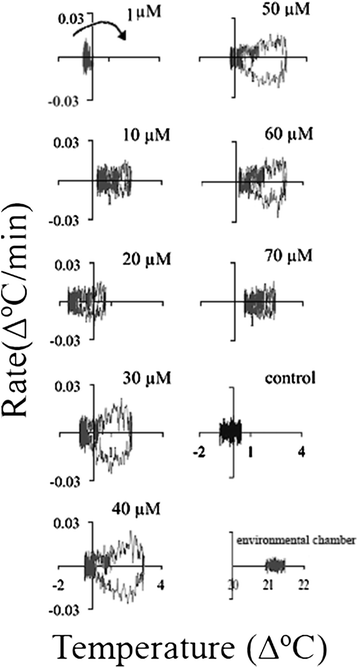

Supplement: Supplementary file 11 — Authors’ original file for figure 11 [file 40529_2014_9068_MOESM11_ESM.gif]

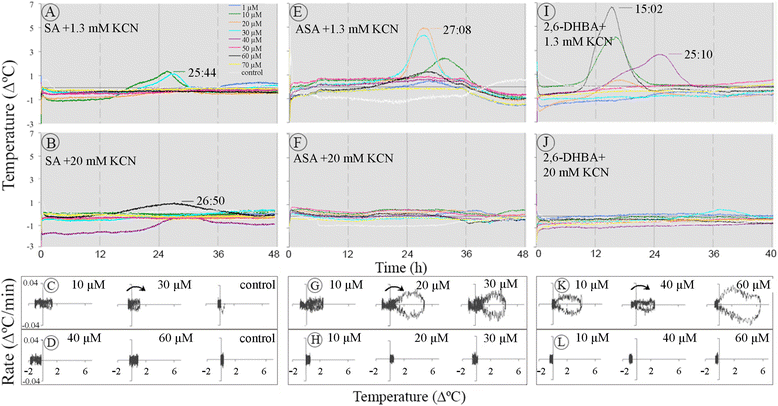

Supplement: Supplementary file 12 — Authors’ original file for figure 12 [file 40529_2014_9068_MOESM12_ESM.gif]

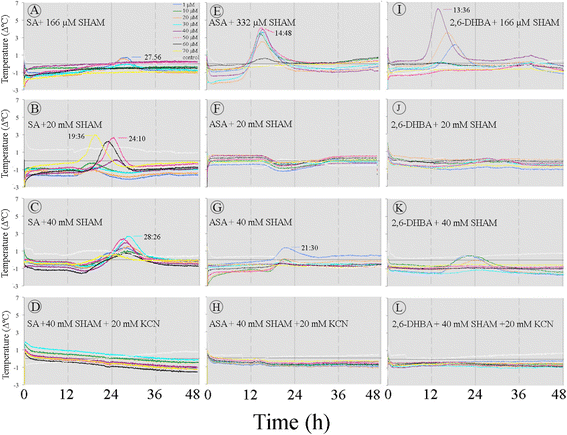

Supplement: Supplementary file 13 — Authors’ original file for figure 13 [file 40529_2014_9068_MOESM13_ESM.gif]

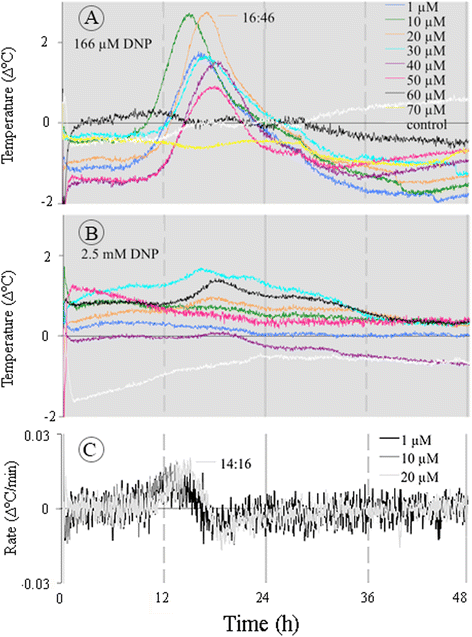

Supplement: Supplementary file 14 — Authors’ original file for figure 14 [file 40529_2014_9068_MOESM14_ESM.gif]

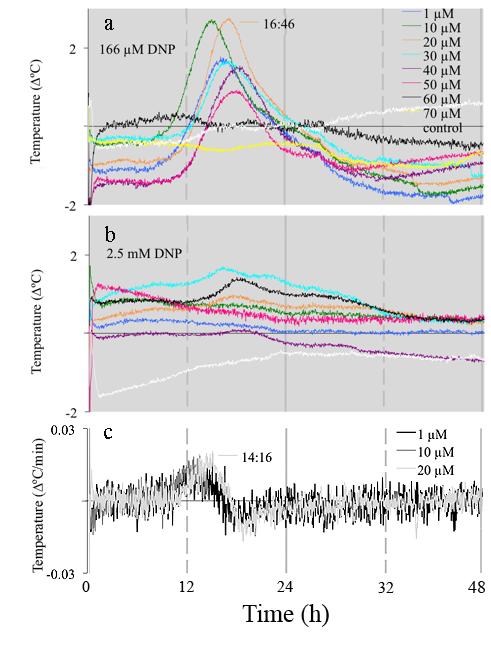

Supplement: Supplementary file 15 — Authors’ original file for figure 15 [file 40529_2014_9068_MOESM15_ESM.tiff]
